# Editorial Brevities

**Published:** 1888-06

**Authors:** 


### EDITORIALS AND MISCELLANEOUS.

### EDITORIAL BREVITIES.

DOCTOR'S PUFFS.—Newspaper notices and puffs of medical men are getting to be quite frequent these times. "He that tooteth not his own horn, verily it shall not be tooted."

SOUTHERN MEDICAL COLLEGE, ATLANTA, GA.—We invite special attention to the advertisement of this institution in this number. It is now fully equipped for the instruction of both medical and dental students.

DR. DEWELL GANN has removed from Bolton, Ga., to Sheridan, Arkansas; having associated himself in practice with Dr. B. W. Mason, of that place. We wish the doctor much success in his new field.

IMPORTANT INCOMPATIBILITY.—The fact is published that the chlorate of potassium and the iodide of iron are incompatible and cannot be combined in prescription without serious resu.ts to the patient.

OIL OF PEPPERMINT is coming into notice as an antiseptic. One drop placed under a bell jar containing a cultivation of cholera bacilli destroys them in a short while. It is said to be death to cockroaches and other insects.

MEDICAL ASSOCIATION OF ALABAMA.—The officers elect for the Alabama Association for the ensuing year are Dr. C. M. Baldridge, President; Dr. B. F. Cross, Jr., Vice-President. The other officers hold over.

THE death of Dr. C. R. Agnew, of New York, which occurred on 18th ult., has called forth numerous expressions of sympathy and tributes to his honor, not only at his home in New York, but elsewhere throughout the country and in Europe.

WHY IS IT?—Will Scudder, I. J. M. Goss, or some other apostle of Eclecticism tell us why it is that *hydrastis*, *euonymon*, *leptandrin*, *caulophyllum*, *viburnum*, *etc.*, are remedies of such prodigious efficacy in the hands of Eclectics whilst the Regulars find them to be medicines of ordinary and limited powers?

INOCULATION AGAINST YELLOW FEVER.—Dr. Geo. M. Sternberg, who was sent by a special Act of Congress to investigate the claim by Dr. Friere, of the discovery of the germ of yellow fever, and of protection from the same by inoculation, has decided that no such discovery has been made, and that inoculation at Rio Janero and Vera Cruz is a failure. But this matter remains to be tested, as it should be, under proper supervision, in localities where yellow fever prevails in this country.

DR. JNO. THAD. JOHNSON.—This eminent and popular member of the profession, so well and favorably known as a Lecturer on Anatomy in the

Atlanta Medical College, and afterwards on Surgery in the Southern Medical College, Atlanta, having removed from the State on account of bad health has returned to Atlanta with his health restored, and has been appointed to the Chair of Operative Surgerv in the Southern Medical College. This is a wise appointment and gives additional strength to this young, vigorous and rapidly rising institution.

TEXAS HEALTH JOURNAL.—Dr. J. R. Briggs, formerly one of the editors of the *Texas Courier-Record of Medicine*, will commence the publication of a journal to be called the *Texas Health Journal*, the first number to appear on the first of July next. We wish Dr. Briggs success in his new enterprise. He has a wide and extensive department, interesting both to the physician and the non-professional reader, and is eminently qualified to edit and conduct such a journal. Price, $2.00 per annum. Addres Richard Flood, Business Manager, Dallas, Texas.

THE LOUISIANA STATE MEDICAL SOCIETY.—Met at Monroe, La , on 25th of April. The officers elected for the year are Dr. I. J. Newton, Jr., President; Vice Presidents—First District, Dr. D. R. Fox, Jesuit's Bend; Second District, Dr. A. G. Fredricks, New Orleans; Third District, Dr. W. D. White, Abbeville; Fourth District, Dr. J. W. Allen, Shrieveport; Fith District, T. C. Brewer, Monroe; C. J. Ducote, Cottonport. Corresponding Secretary, Dr. A. A. Lyon, Shreveport; Orator, Chas. A. Boatner, Shrieveport. The next place of meeting will be New Orleans, the second Tuesday in April, 1889.

A LIBERAL OFFER.—We have an arrangement by which any of our readers who will send us the name of a new subscriber, with $2.00 inclosed, will receive a copy of the *New York World* (weekly) from now until the close of the presidential canvass, or until the 13th of November, next. The approaching presidential election will be one of intense and unusul excitement and interest, and our readers have here an easy method of obtaining a copy of the leading Democratic paper in the United States, which will keep them well posted in the great political conflict now opening.

A NEW FOREIGN HUMBUG COMING.—The editor of *National Druggist* in referring to the gratuitous advertising which our medical journals are giving to foreign "antiseptins, antipyrins, and other antis," invites attention to Gawalowski's "merciless exposure of a new compound which is getting ready in Germany to make a descent on Europe and America in the style of its predecessors—the antiseptic kreolin, of the wondrous value of which the advance guard of certificates have already commenced to appear in our journals. Will the latter be warned in time, or will they swindle themselves out of thousands of dollars by giving it the usual American welcome and gratis advertising?

WM. R. WARNER & CO.—Mr. H. M. Pinkard, the gentlemanly agent of that staunch and reliable house of Wm. R. Warner & Co., called upon us recently with beautiful samples, amongst them his excellent preparation of bromo soda, so admirable as a remedy for headaches and nervous conditions, podophyllin parvules, so fine in constipation and liver troubles, his pill digestiva, so useful in dyspepsia, etc. In addition to these we are pleased to acknowledge a beautiful and unique gift in the shape of a knife ("ingluven knife") for trimming, opening envelopes and like purposes, very acceptable, and always on our editorial table, it will prove not only an implement of usefulness, but a pleasant reminder of our warmly esteemed friends and patrons, Wm. R. Warner & Co.

